# Systematic Review of the Impact of COVID-19 on Healthcare Systems and Society—The Role of Diagnostics and Nutrition in Pandemic Response

**DOI:** 10.3390/jcm14072482

**Published:** 2025-04-04

**Authors:** Wanda Olesińska, Małgorzata Biernatek, Sabina Lachowicz-Wiśniewska, Jacek Piątek

**Affiliations:** Faculty of Medicine and Health Science, University of Kalisz (Calisia University), plac Wojciecha Bogusławskiego 2, 62-800 Kalisz, Poland; wandaole@wp.pl (W.O.); m.biernatek@uniwersytetkaliski.edu.pl (M.B.); j.piatek@uniwersytetkaliski.edu.pl (J.P.)

**Keywords:** healthcare, nutrition, COVID-19, immune response, pandemic management, mental health

## Abstract

The COVID-19 pandemic has revealed deep vulnerabilities in healthcare systems and public health preparedness. This systematic review examines the effectiveness of epidemiological procedures, the role of diagnostics, and the influence of nutritional status on immune function and disease severity. A total of 88 studies were analyzed, encompassing diagnostics, micronutrient deficiencies (notably vitamin D, C, E, zinc, and selenium), and the psychosocial impact of the pandemic. The results underscore the importance of integrated strategies—including accurate testing, preventive nutritional measures, and mental health support—in improving outcomes and societal resilience during global health crises. Unlike previous reviews that focused on isolated biomedical or public health elements, this study integrates diagnostics, immune-nutritional status, and psychosocial effects to present a comprehensive, multidimensional analysis of pandemic impact and preparedness.

## 1. Introduction

The COVID-19 pandemic, caused by the SARS-CoV-2 virus, has emerged as one of the most significant health challenges of the 21st century, affecting nearly every aspect of social, economic, and medical life worldwide. The rapid spread of the virus, high infection and mortality rates, and the emergence of new pathogen variants necessitated the implementation of effective epidemiological procedures and the reorganization of healthcare systems. Simultaneously, the pandemic underscored the importance of diagnostics, nutritional status, and immune system resilience in combating infections while also highlighting the severe psychosocial consequences resulting from isolation and restrictions [1,2,3].

Preventive measures, such as quarantine, isolation, testing, and vaccination campaigns, were introduced to curb viral transmission and ensure the proper functioning of healthcare systems. At the same time, attention was drawn to the significance of nutritional status, including the role of vitamins and minerals (D, C, E, and zinc) in modulating immunity and mitigating the severity of COVID-19 infections. As the pandemic situation evolved dynamically, its negative social and psychological effects became evident, affecting a significant portion of the population and leading to increased levels of stress, anxiety, and other mental health disorders [4,5].

Vitamin D plays a crucial role in modulating immune function, influencing both the innate and adaptive immune systems. It has been shown to regulate the expression of pro-inflammatory and anti-inflammatory cytokines, which are key players in immune response. Additionally, vitamin D is involved in the regulation of the renin-angiotensin system, which may contribute to reducing the inflammatory response associated with viral infections. Emerging research suggests a possible link between vitamin D levels and the severity of COVID-19 outcomes, making it a topic of increasing interest in public health strategies [6,7].

Therefore, the primary objective of this study was to analyze and assess the effectiveness of epidemiological procedures implemented during the COVID-19 pandemic, considering their impact on the healthcare system, society, and individual health. The study focuses on the importance of diagnostics, the role of nutritional status in shaping immune resilience, and the psychosocial consequences of the pandemic. A precise examination of these aspects aims to identify key measures essential for effective crisis management in the future. While several studies have separately addressed the medical or social aspects of the COVID-19 pandemic, there remains a lack of integrative reviews combining diagnostics, nutritional immunity, and societal responses. This review aims to fill that gap by synthesizing evidence across these areas to support future multidimensional pandemic response strategies.

## 2. Methodology Review

### 2.1. Literature Review

**Figure 1:**
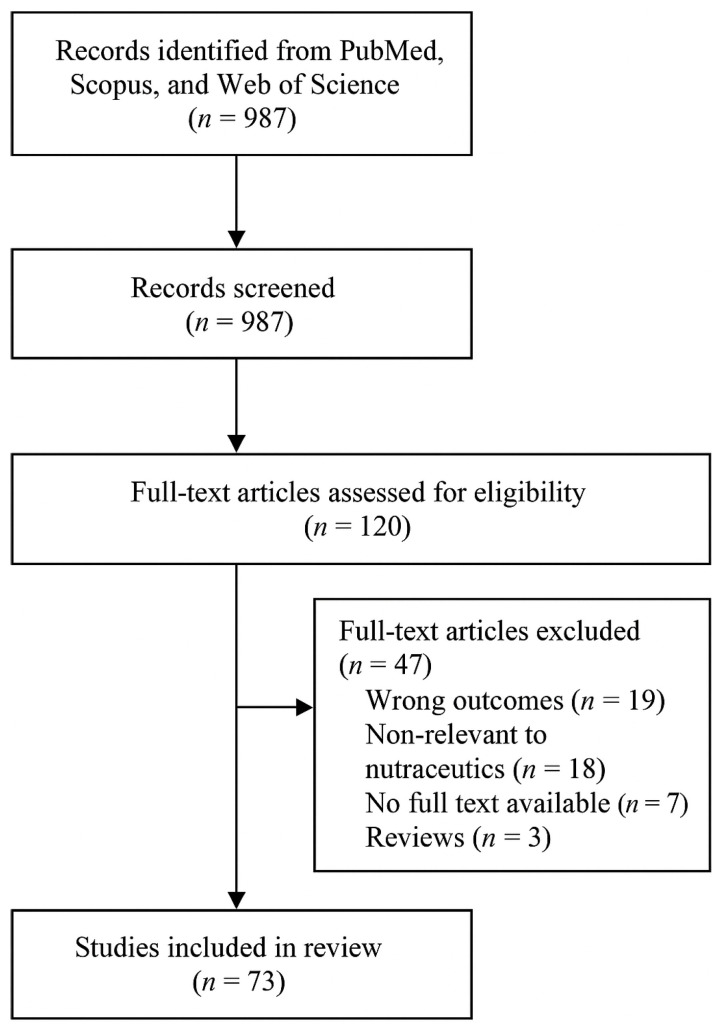
PRISMA Flow Diagram of the Study Selection Process.

A comprehensive literature search was conducted in scientific databases, including Science Direct (Elsevier), Springer Link, EBSCO, Nature, Science, Wiley Online Library, Scopus, and Web of Science, covering publications available up to October 2024. The search focused on key terms related to the COVID-19 pandemic and the analyzed areas, such as epidemiological procedures, COVID-19 diagnostics, the impact of the pandemic on public health, immune system resilience, nutritional components, mental health, social consequences of the pandemic, and health protection strategies. Additionally, reference lists of included studies and relevant review articles were examined to identify further publications. This review follows the PRISMA (Preferred Reporting Items for Systematic Reviews and Meta-Analyses) guidelines for systematic reviews, ensuring transparency in literature selection and synthesis. A PRISMA flowchart detailing the selection process is included in Figure 1.

Several studies have explored the relationship between vitamin D levels and COVID-19 severity. A study conducted by Radujkovic et al. [8] found that mortality was significantly higher (21%) in hospitalized patients with vitamin D deficiency compared to those with sufficient levels (3.1%) [9]. Additional observational research supports a link between deficiency and increased symptom severity or prolonged hospitalization [10]. However, while these findings suggest a correlation, results from randomized controlled trials (RCTs) remain inconsistent. For instance, Murai et al. [11] reported no significant improvement in hospital stay duration after high-dose vitamin D supplementation [12]. Meanwhile, other RCTs suggest modest benefits in respiratory outcomes. These mixed results emphasize the need for more rigorously designed trials to clarify the clinical value of vitamin D in COVID-19 prevention and treatment [13].

### 2.2. Selection Criteria

The analysis included studies that met the following criteria: (1) focused on the impact of the COVID-19 pandemic on public health, the healthcare system, and society; (2) examined epidemiological procedures, diagnostics, and the significance of immune resilience, including the role of nutritional components (vitamins and minerals); (3) considered biological mechanisms related to immune system function and the psychosocial consequences of the pandemic; (4) based on experimental, observational, or review-based research approaches; (5) published in English or Polish. Studies were excluded if they (1) did not provide clear definitions of the analyzed phenomena (e.g., lacked descriptions of diagnostic procedures or epidemiological measures); (2) did not address the impact of COVID-19 on public health or immune function; (3) focused solely on pharmacological interventions unrelated to immunity or pandemic effects; (4) examined populations with diseases that could compromise results unrelated to the analyzed areas. The restriction to English and Polish language publications was based on the authors’ language proficiency and the relevance of Polish national data to the analyzed topics. In total, 87 articles were included in the final analysis. Among the 75 included studies, the majority originated from Poland (*n* = 28), followed by the United States (*n* = 14), the United Kingdom (*n* = 9), Italy (*n* = 6), and other European countries (*n* = 15). Of these, 18 focused on nutritional aspects (e.g., vitamins and minerals), 14 examined immunological mechanisms, 16 addressed diagnostic approaches, and 24 investigated biopsychosocial or systemic impacts of the pandemic. The remaining studies contributed to broader epidemiological or organizational themes not fully captured by these four predefined categories.

## 3. Results

The first case of COVID-19 infection worldwide was recorded in late 2019. The first confirmed case in Poland was diagnosed on 4 March 2020. Available publications suggest that the coronavirus emerged in the last quarter of 2019 in China and exhibited similarities to two other coronaviruses that caused epidemics in 2003 and 2012. A common pathological feature of all these viruses was rapid lung involvement and the development of massive acute respiratory failure. Coronaviruses constitute a diverse group of viruses that can infect various animal species and cause mild to severe respiratory infections in humans [1].

This situation often required rapid oxygen administration and, in severe cases, mechanical ventilation. In the most critical cases, extracorporeal membrane oxygenation (ECMO) was used to oxygenate the blood externally. SARS-CoV-2 quickly spread globally, resulting in a surge in infections [2] and deaths [3]. As a consequence of the drastic increase in mortality rates due to SARS-CoV-2 infections, the World Health Organization (WHO) declared a pandemic. From that moment, numerous researchers made intensive efforts to understand and describe the mechanisms of COVID-19, ultimately leading to the development of prevention and treatment strategies. Over time, vaccines against the virus became available, along with potential antiviral drugs aimed at inhibiting viral replication within human cells.

## 4. Infectivity and Diagnostics of SARS-CoV-2

The SARS-CoV-2 coronavirus belongs to the Coronaviridae family, including viruses that infect humans and animals. It is characterized by the presence of a spike (S) protein, which enables the virus to bind to the ACE2 (angiotensin-converting enzyme 2) receptor located on the surface of host cells, a crucial step in the infection process. SARS-CoV-2 exhibits a high mutation rate, leading to the emergence of multiple variants, such as Delta and Omicron, which differ in infectivity and immune evasion capabilities. The high transmissibility of the virus and the diverse modes of transmission, including droplet infection, contact with contaminated surfaces, and aerosols, contributed to its rapid global spread [5]. Understanding the structure and mechanism of action of the coronavirus was essential for the development of effective diagnostic methods. Diagnosis primarily relied on RT-PCR (reverse-transcription–polymerase chain reaction or reverse-transcriptase–polymerase chain reaction) tests, which detected the viral genetic material, as well as rapid antigen tests, particularly for symptomatic patients. Accurate identification of the virus was necessary for monitoring the progression of the pandemic, detecting new infection clusters, and evaluating the effectiveness of implemented epidemiological and therapeutic procedures.

### 4.1. Modes of Transmission

SARS-CoV-2 infection can occur through various transmission routes:

- Droplet transmission—the primary mode of human-to-human infection;
- Contact with contaminated surfaces—everyday objects on which the virus has settled, enabling its transfer to the oral and nasal cavities;
- Fecal–oral transmission—via saliva, urine, or feces;
- Ocular transmission—through tears and conjunctival secretions;
- Bloodborne transmission—via direct contact with infected blood [5].

It is well established that individuals of all ages are susceptible to SARS-CoV-2 infection, with disease severity varying according to age. The mildest and least severe cases are observed in children, whereas the most severe cases predominantly occur in individuals over 65 years of age. Middle-aged individuals often present with chronic conditions associated with aging, while stress and other factors may exacerbate inflammatory states. In elderly populations, these chronic diseases and inflammatory processes tend to intensify further.

SARS-CoV-2 infection progresses relatively rapidly, with symptom onset typically occurring 2 to 3 days after exposure to an infected individual. The peak infectivity is observed on the fifth-day post-exposure, at which point the infected person poses the greatest risk of transmission to others in their surroundings [14].

Until November 2021, the Delta variant was designated as a variant of concern (VOC) due to its distinctive characteristics. According to the Centers for Disease Control and Prevention (CDC), Delta exhibited increased transmissibility, more severe disease course, reduced treatment efficacy, and other concerning properties [15].

The Omicron variant (B.1.1.529) is a highly mutated SARS-CoV-2 variant that was classified by the WHO as a VOC on 26 November 2021 [16]. The first confirmed case of Omicron infection was derived from a sample collected on 9 November 2021 in South Africa, which was subsequently reported to WHO on 24 November 2021. Since then, numerous cases of Omicron infection have been identified globally [17].

The WHO Technical Advisory Group on SARS-CoV-2 Virus Evolution (TAG-VE) identified Omicron as a divergent variant of SARS-CoV-2, distinguished by 26 to 32 mutations in the spike (S) protein [18]. These mutations affect critical regions, including the receptor-binding domain (RBD) and the N-terminal domain (NTD), potentially facilitating enhanced viral entry into host cells, increased transmissibility, and immune evasion.

The Omicron variant comprises four Pango lineages: B.1.1.529, BA.1, BA.2, and BA.3 [19]. Molecular diagnostic assays, particularly polymerase chain reaction (PCR)-based genomic tests, detect the 69–70 spike protein deletion, leading to a phenomenon known as S-gene target failure (SGTF), which serves as a distinguishing marker for Omicron detection [20].

### 4.2. Types of Diagnostic Tests and Their Effectiveness

According to the WHO, in regions experiencing community transmission of COVID-19, the detection of a single viral gene is sufficient to confirm infection. In Poland, under the official case definition for COVID-19 as of 31 October 2020, identifying a single SARS-CoV-2 gene meets the laboratory criteria for confirming infection. However, due to the risk of false-negative results associated with emerging viral variants, WHO recommends the use of diagnostic tests detecting at least two or more fragments of the SARS-CoV-2 genome [16]. Consequently, in Poland, dual-target assays are advised to be used, with optimal detection covering three or more genomic regions of the virus.

Genetic testing requires appropriate laboratory validation and verification, and test results must be interpreted following manufacturer guidelines. A positive RT-PCR result confirms SARS-CoV-2 infection, whereas an uncertain result neither confirms nor excludes infection, necessitating a repeat test with a new sample collected after 24–48 h. A single negative RT-PCR result indicates a low probability of infection and generally does not require confirmation except in specific situations outlined in clinical recommendations.

In symptomatic patients, a repeat RT-PCR test is recommended in the following cases:

(a) High probability of infection based on clinical presentation, epidemiological history, or lung imaging findings (retesting recommended within 24–48 h).
(b) Worsening respiratory symptoms warranting an additional RT-PCR test (within 24–48 h of the first test).
(c) For intubated patients, testing on lower respiratory tract samples may be considered.

Additionally, retesting is mandatory if the initial test was improperly performed, such as incorrect sample collection or storage; in such cases, the test should be repeated immediately [21].

Apart from RT-PCR, serological tests have been developed to detect SARS-CoV-2 antibodies. These methods analyze clinical samples in combination with specific viral antigens to identify patient immune responses, aiding epidemiological studies on COVID-19. The primary objective of serological testing is to provide qualitative or semi-quantitative assessment of antibody levels, using techniques such as (Table 1):

(1) ELISA (enzyme-linked immunosorbent assay)—immobilized antigen proteins bind to target antibodies on a microplate surface.
(2) CLIA (chemiluminescent immunoassay)—combines immunochemical reactions with chemiluminescent detection.
(3) LFIA (lateral flow immunoassay)—employs lateral flow technology for rapid antibody detection.

Among these, LFIA is considered one of the most promising techniques due to its low technical requirements, affordability, reduced sample preparation risks, and high sensitivity and specificity. Moreover, LFIA enables result acquisition within 15 min [22,23].

Serological tests are categorized into the following:

(1) Antigen detection tests—identifying viral protein fragments either on the virus surface or internally, allowing active infection detection within 15 min compared to the several hours required for RT-PCR.
(2) Antibody detection tests—measuring immunoglobulin (IgG and IgM) levels in blood, serum, or plasma to determine whether an individual is actively fighting an infection or has prior exposure to SARS-CoV-2.

Two main diagnostic methods for antigen detection include the following:

(a) ICT (immunochromatographic test)—utilizes colloidal gold-conjugated antibodies to create visible color bands indicating a positive result.
(b) FIA (fluorescent immunoassay)—employs an automated immunofluorescence reader for test interpretation [24].

Serological testing plays a crucial role in epidemiological studies but is less specific than RNA-based SARS-CoV-2 detection methods. Systematic reviews have analyzed multiple studies comparing different serological tests for COVID-19 diagnosis. Some assays detect individual antibody classes (IgG, IgM, or IgA), while others analyze combinations of immunoglobulin types. Specificity and sensitivity vary across test types, and their effectiveness depends on the disease stage, as antibody levels fluctuate throughout the infection.

Compared to RT-PCR, serological tests offer faster analysis times, reduced sample handling risks, and lower technical requirements. They complement RT-PCR in asymptomatic patient screening, contributing to enhanced epidemiological surveillance and improved COVID-19 diagnostics [25].

Beyond RT-PCR and serological testing, there is growing interest in next-generation biosensors for rapid, reliable, and highly sensitive COVID-19 detection. Novel biosensors for SARS-CoV-2 detection include:

(1) ACE-2 receptor-based biosensors for detecting virus binding activity.
(2) Gold nanoparticle-based biosensors for enhancing signal detection.
(3) FET (field-effect transistor) biosensors, offering exceptional sensitivity and real-time detection.
(4) ROS (reactive oxygen species)-based biosensors, enabling rapid, cost-effective, and highly sensitive viral detection.

Among these, FET-based biosensors are particularly promising, as they offer fast and highly sensitive virus detection. Similarly, ROS-level detection biosensors demonstrate potential for low-cost, rapid diagnostics. However, scaling up the production and distribution of these biosensors remains a significant challenge, limiting their widespread implementation in medical settings.

Additionally, electromagnetic biosensors have emerged as a novel diagnostic approach, providing high sensitivity for SARS-CoV-2 detection at point-of-care facilities.

Rapid antigen tests can be utilized for the diagnosis of symptomatic SARS-CoV-2 infections within the first seven days after symptom onset. Symptomatic individuals with a positive antigen test result should be considered infected, whereas a negative result does not exclude infection and requires confirmation via RT-PCR. An initial antigen test may be conducted for asymptomatic individuals at risk of infection due to documented exposure to a confirmed COVID-19 case—particularly in outbreak settings. Individuals testing positive should be considered infected, while those with negative results should undergo genetic testing to confirm or exclude infection. Rapid antigen tests may be used at the point of care (POCT) if the test manufacturer has validated such use [21]. Since October 2021, in addition to rapid antigen tests for COVID-19, the Health Security Committee (HSC) has approved a list of mutually recognized laboratory-based antigen tests, including enzyme-linked immunosorbent assays (ELISA) and automated immunoassays for antigen detection. Although these devices met the same criteria as rapid antigen tests, they were not eligible for issuing EU Digital COVID Certificates (EU DCC) until 30 June 2022. The first list of mutually recognized laboratory-based antigen tests for COVID-19 was adopted by the Health Security Committee on 20 October 2021, with subsequent updates on 10 February, 8 April, and 10 June 2022 [26].

## 5. Epidemiological Procedures

### 5.1. National and International Guidelines

On 2 July 2020, the Polish Ministry of Health issued an updated set of guidelines for primary healthcare (PHC) nurses during the SARS-CoV-2 epidemic. These guidelines were designed to enhance safety protocols and facilitate the work of nurses providing primary healthcare services. Given the need for individualized patient care, the general principles were published on the Ministry’s website.

The guidelines focused on limiting in-person visits in both home settings and nursing offices, promoting teleconsultations as the primary mode of patient contact. In cases where a home visit was necessary, it had to be arranged in advance via telephone. Patients were informed about the possibility of receiving an e-prescription code or information on medical supplies via SMS, email, or phone call. Before conducting a home visit, nurses were required to conduct a preliminary phone interview with the patient or their family and check the e-WUŚ system to verify whether the patient was under quarantine restrictions [27].

The Ministry of Health also introduced detailed occupational health and safety (OHS) regulations concerning preventive medical examinations for healthcare personnel and safety training protocols for various healthcare sectors, including dentistry, palliative and hospice care, primary care nursing, long-term care, pediatrics, psychiatry, addiction treatment, hematology, translatology, oncology radiotherapy, diabetology, perinatal care, emergency medical services, ophthalmology, and rehabilitation [28,29].

These guidelines provided clear procedural instructions for different healthcare settings, significantly facilitating the work of medical personnel during the pandemic. Despite the availability of epidemiological guidelines, specific legal regulations apply when handling patients suspected of SARS-CoV-2 infection, including the following:

- The Act on the Prevention and Control of Infections and Infectious Diseases in Humans [30];
- The Minister of Health’s Regulation of 8 October 2020 regarding organizational standards for healthcare services for patients suspected or confirmed to have SARS-CoV-2 infection [31];
- The Minister of Health’s Regulation of 12 August 2020 on organizational standards for teleconsultation in primary healthcare [31];
- The Minister of Health’s Regulation of 5 March 2021 amending the organizational standards for teleconsultation in primary healthcare [29].

The Act on the Prevention and Control of Infections and Infectious Diseases in Humans defines the principles and procedures for infection prevention, control, and monitoring in Poland. It outlines the responsibilities of public health authorities, the rights and obligations of healthcare providers, and infection control measures to minimize the spread of infectious diseases and immunize vulnerable populations [31].

Additional epidemic safety guidelines were issued by the Chief Sanitary Inspectorate (GIS). On 10 June 2022, updated health and safety recommendations were published on gov.pl, outlining protective measures for preschools, early education units, and childcare institutions for children under three years old. These recommendations were based on Article 8a, Section 5, Point 2 of the 14 June 1985 Act on the State Sanitary Inspection [32,33].

The document covered critical areas, including the following:

- Facility organization and safety protocols;
- Hygiene and sanitation;
- Cleaning and disinfection procedures;
- Gastronomy-related safety measures;
- Protocols for suspected infections among staff or children.

School and preschool directors were authorized to define additional internal safety measures within their institutional regulations. The document also addressed daily hygiene practices, such as the following:

- Handwashing protocols;
- Child drop-off and pick-up procedures;
- Clothing hygiene standards;
- Sector-Specific Guidelines for the Food Service Industry.

On 13 May 2020, the Chief Sanitary Inspectorate (GIS) also issued sector-specific epidemic guidelines for the food service industry, available on gov.pl. These guidelines were divided into four main categories:

- Employee safety measures;
- Customer safety measures;
- Preventive procedures for suspected infections among employees;
- Procedures for handling suspected infections in customers [33,34].

These comprehensive guidelines were crucial in minimizing COVID-19 transmission risks across various healthcare and public service sectors, ensuring a structured approach to pandemic response and workplace safety.

### 5.2. Organization of Healthcare During the Pandemic

The Hospital Infection Control Team report highlighted essential SARS-CoV-2 prevention measures. At the onset of the pandemic in 2020, a procedure titled “Management of Individuals Suspected or Confirmed to Have SARS-CoV-2 Who Independently Reported to the County Hospital in Jarocin” was implemented. Visitation restrictions were introduced, allowing visits only with the approval of department heads, and by March 2020, an absolute visitation ban was enforced.

A separate pathway for patients with respiratory infectious disease symptoms was established within the Emergency Department (ED), featuring a dedicated entrance to an isolation unit. Patients with acute respiratory infections were directed to an isolation zone marked with a poster indicating “ACUTE RESPIRATORY INFECTIONS”.

By March 2020, the procedure was updated, introducing mandatory body temperature measurements for hospital staff, particularly upon entering the hospital premises. Additionally, planned admissions and outpatient clinic operations were reduced, as well as hospital diagnostic activities. A sanitary protocol was implemented for laboratory sample collection, including social distancing measures and protective equipment for staff. By the end of March, specific protocols for emergency cesarean sections and urgent trauma procedures were introduced.

A mandatory epidemiological statement regarding their SARS-CoV-2 exposure history was enforced for all hospital service users. Isolation rooms were designated for suspected COVID-19 patients, including delivery rooms and intensive care unit (ICU) stations. Throughout the pandemic, hospital staff received regular updates on new guidelines, which were also made available online. Additional training sessions, workplace briefings, and instructions were provided, including workshops on personal protective equipment (PPE) use by the hospital’s occupational health and safety (OHS) inspector.

By mid-April 2020, the use of PPE was mandated for staff when dealing with both symptomatic and asymptomatic patients. By the end of April, serological screening tests were introduced for patients. SARS-CoV-2 tests were processed at the Virology Laboratory of WSSE in Poznan, with 75 urgent tests (throat and nasal swabs) conducted via REAL-TIME PCR between 4 March and 30 April 2020.

In early May 2020, a directive was issued regarding COVID-19 diagnostic testing for medical and hospital staff, alongside a mandatory patient statement on the risk of SARS-CoV-2 infection before undergoing surgical procedures. The hospital continuously monitored PPE stock levels, ensuring availability in both the hospital pharmacy and additional supply points. Hand sanitization stations were installed at every hospital entrance, hallway, and information desk, accompanied by hand hygiene posters and instructional diagrams.

Hospital management closely followed the recommendations of the Ministry of Health (MZ), the Chief Sanitary Inspectorate (GIS), and national consultants on SARS-CoV-2 prevention, adapting protocols accordingly. Legal regulations were continuously monitored, and recommendations were implemented based on government policies. In mid-September 2020, the hospital established a sampling point for RT-PCR COVID-19 testing, available under public health insurance (NFZ) and commercial services [35].

Pandemic response procedures evolved over time. In the early phase, travel restrictions were imposed, borders, airports, and ports were closed, and quarantine and isolation were mandated for travelers from high-risk areas. Handwashing recommendations and facial contact avoidance guidelines were widely disseminated. Mass gatherings were banned [35].

Subsequent phases involved social restrictions and lockdowns, during which social isolation measures were introduced in many countries. Schools, restaurants, and public venues were closed, and mandatory face masks were enforced in designated regions. Where possible, remote work was implemented, and public movement was strongly discouraged. Additionally, testing and contact tracing efforts intensified [36].

As restrictions gradually eased, public spaces reopened while maintaining social distancing guidelines, mandatory mask usage in selected areas, and continued testing and contact tracing [36,37].

The emergence of the second and third pandemic waves led to the reintroduction of restrictions and new protective measures, including increased testing and expanded vaccine access. By early 2021, many countries had launched mass vaccination campaigns and established specialized vaccination centers [37].

As economies reopened and restrictions were lifted, emerging virus variants necessitated ongoing vaccine adaptations. Between late 2021 and early 2022, health passports were introduced, allowing vaccinated individuals or those with negative test results to participate in public activities [38].

Expert recommendations on epidemiology covered multiple areas, including diagnostics, disease progression, and clinical monitoring. The first level refers to asymptomatic or mildly symptomatic patients, who often present with no symptoms or mild upper respiratory tract discomfort (fever, cough without dyspnea), sometimes accompanied by headaches, muscle pain, nausea, vomiting, and diarrhea. Hemoglobin oxygen saturation measured by pulse oximetry (SpO2) is ≥94%; the clinical condition remains stable. Diagnostics included considerations regarding influenza testing, depending on the current epidemiological situation. The patient does not require imaging or biochemical tests. In cases of persistent cough and/or symptoms indicating lower respiratory tract involvement, appropriate imaging tests are recommended. A patient in this condition does not require hospitalization but rather treatment in isolation. During isolation, it is recommended to assess the general condition twice daily and measure temperature, heart rate, and blood pressure [21,35,39].

The second level refers to symptomatic patients with pneumonia without signs of respiratory failure. Symptoms often include fatigue, asthenia, fever > 38 °C, cough, dyspnea, and other extrapulmonary symptoms. Clinical and radiological signs of lung involvement are present. Due to the risk of clinical deterioration, the patient requires monitoring and interventions to accelerate the elimination of SARS-CoV-2 infection. No clinical or laboratory signs of respiratory failure are present (SpO2 < 94% but greater than 90%). Diagnostics included influenza testing depending on the epidemiological situation and/or other respiratory pathogens as determined by the clinical context. In cases of persistent fever >38 °C, blood cultures should be performed. Laboratory tests included complete blood count with differential, platelet count, C-reactive protein (CRP), procalcitonin, glucose, creatinine, alanine aminotransferase (ALT), bilirubin, lactate dehydrogenase (LDH), prothrombin time (INR), D-dimer, cardiac troponins, and IL-6 assessment (detailed recommendations below). Hospitalization is required due to the risk of disease progression. Hospital monitoring includes temperature, blood pressure, heart rate, respiratory rate, and pulse oximetry (2–3 times daily). Arterial blood gas analysis and acid–base balance assessment should be performed, particularly on days 5–7 after symptom onset or in cases of sudden clinical deterioration [35,36].

The third level refers to patients with severe pneumonia and respiratory failure. Clinical symptoms include worsening respiratory function and gas exchange impairment (dyspnea, respiratory rate > 30/min, SpO2 < 90%). This phase is characterized by silent hypoxia. The patient exhibits acute respiratory symptoms requiring strict monitoring, particularly between days 5 and 7 after symptom onset, to assess the need for intensive medical care. Imaging studies typically show lung involvement > 50% of the pulmonary parenchyma [35]. Possible extrapulmonary manifestations include ischemic or hemorrhagic strokes, deep vein thrombosis and/or encephalitis, Guillain–Barré syndrome, altered consciousness, and seizures. Cardiovascular complications may include ischemic heart disease, myocarditis, and arrhythmias [21,37,38]. There are no signs of ARDS (acute respiratory distress syndrome), septic shock, multiple organ failure, or altered consciousness. Diagnostics included influenza testing depending on the epidemiological situation and/or other respiratory pathogens, as determined by the clinical context. In cases of persistent fever > 38 °C, blood cultures should be performed. Further diagnostics should be conducted depending on the clinical presentation (e.g., HIV testing). Laboratory tests included complete blood count with differential, platelet count, CRP, procalcitonin, arterial blood gas analysis with acid–base balance assessment, glucose, ferritin, IL-6, creatinine, elevated serum aspartate aminotransferase (AST), elevated serum alanine aminotransferase (ALT), amylase, albumin, bilirubin, lactate dehydrogenase (LDH), lactate, INR, D-dimer, cardiac troponins, B-type natriuretic peptide (BNP), and NT-proBNP (detailed recommendations below). Imaging studies are outlined in the Diagnostic Imaging Recommendations section. Strict clinical monitoring and assessment of vital signs (temperature, blood pressure, heart rate, respiratory rate, Glasgow Coma Scale, SpO2) should be conducted. Arterial blood gas analysis and acid–base balance assessments are necessary. Echocardiography is recommended in cases of suspected acute heart failure.

The fourth level refers to ARDS/multiple organ failure. The patient is in critical condition, with respiratory failure and impairment of other vital functions: acute respiratory distress syndrome (ARDS), sepsis, septic shock, and multiple organ failure. The Berlin definition categorizes ARDS into three severity levels: mild (200 mmHg < PaO2/FiO2 ≤ 300 mmHg with PEEP or CPAP ≥ 5 cmH_2_O, or in non-ventilated patients); moderate (100 mmHg < PaO2/FiO2 ≤ 200 mmHg with PEEP ≥ 5 cmH_2_O in non-ventilated patients); and severe (PaO2/FiO2 ≤ 100 mmHg with PEEP ≥5 cmH_2_O) [21,39]. In ICU settings, the risk of arterial or venous thromboembolic events ranges from 31% to 59%. Diagnostics include testing for influenza and other respiratory pathogens responsible for upper respiratory tract infections (aerosol-generating procedures should be avoided due to the risk for healthcare personnel). In cases of persistent fever >38 °C, blood cultures should be performed. Further diagnostics should be conducted based on clinical presentation (e.g., HIV testing). Laboratory tests included complete blood count with differential, platelet count, CRP, IL-6, procalcitonin, arterial blood gas analysis with acid–base balance assessment, glucose, ferritin, creatinine, ALT, AST, amylase, albumin, bilirubin, LDH, lactate, INR, D-dimer, activated partial thromboplastin time (APTT), fibrinogen, cardiac troponins, BNP, and NT-proBNP (detailed recommendations below). Imaging studies are outlined in the Diagnostic Imaging Recommendations section. Strict clinical monitoring and assessment of vital signs in ICU settings are required. Arterial blood gas analysis and acid–base balance assessments are necessary. Echocardiography is recommended in cases of suspected acute heart failure [21].

The Agency for Health Technology Assessment and Tariff System published recommendations for diagnostic procedures in response to the reduced epidemic risk associated with COVID-19 (version 1.1, 7 April 2022). The document outlines guidelines for referring patients with fever and/or respiratory infection symptoms to teleconsultations and in-person visits in primary healthcare, SARS-CoV-2 testing, and sanitary-epidemiological surveillance [39].

#### Challenges Faced by Healthcare Systems During the Pandemic

As mentioned earlier, in December 2019, the first cases of pneumonia of unknown origin were reported in Wuhan, China. On 5 January 2020, scientists successfully sequenced the pathogen, identifying it as a novel coronavirus. Interestingly, in the United States, the first confirmed infection was reported on 21 January 2020 in Seattle, and within 24 h, the virus was also detected in a patient in South Korea. While South Korea immediately began COVID-19 testing using WHO-approved tests, the United States opted to develop its own diagnostic tests through the Centers for Disease Control and Prevention (CDC), but these tests proved faulty. The lack of effective and widely available tests resulted in a nearly 50-day delay before mass testing was implemented in the United States, allowing the virus to spread undetected. During this period, thousands of patients in the USA presented at hospitals, urgent care facilities, and medical offices with pneumonia symptoms and respiratory infections. Without the ability to rapidly diagnose COVID-19 or even suspect the disease, infected patients unknowingly transmit the virus to healthcare workers, family members, and the broader community.

For comparison, by February 2020, South Korea had conducted over 75,000 tests, whereas the United States had performed only 352. South Korea implemented all key WHO recommendations, including mass testing, contact tracing, testing exposed individuals, isolation of confirmed cases, and social distancing measures. Similar strategies were adopted by Singapore and Hong Kong, which emphasized protecting healthcare workers. Medical personnel needed to wear surgical masks during every interaction with patients in these locations.

The United States also faced severe shortages of essential medical equipment at a critical moment. The drastic deficit of masks, personal protective equipment (PPE), ventilators, and other vital resources forced healthcare workers to reuse masks for multiple days. The shortages extended beyond staff—in some intensive care units, multiple patients had to share a single ventilator. At the pandemic’s peak, the demand for ventilators in the USA ranged from several hundred thousand to nearly one million, but fewer than 160,000 were available. The crisis was exacerbated by censorship of healthcare professionals, with many doctors and nurses being silenced, penalized, or even dismissed for publicly voicing their concerns and highlighting the dire conditions in hospitals [4].

One of the exceptional consequences of the pandemic for healthcare workers was the phenomenon of moral injury, which was previously associated primarily with military veterans. However, this issue also affected physicians and emergency medical responders during the pandemic. A moral injury arises when an individual participates in actions that conflict with their moral values, is unable to prevent unethical events, or witnesses such events, leading to profound internal conflict and psychological distress. In the early months of the pandemic, the media extensively reported on the overwhelming burden on New York’s emergency services and the crisis caused by ventilator shortages. Doctors and nurses were faced with heart-wrenching decisions, having to determine who would receive life-saving support and who would be left without it. Although medical personnel are trained to handle situations involving patient deaths, the necessity of withholding treatment from individuals who could have been saved under normal circumstances was an extremely painful and traumatic experience for many [5].

## 6. The Role of Vaccination in Combating the Pandemic—Social Issues

The changes in daily life that occurred during the pandemic were rapid and unprecedented. The sharp increase in new infections and fatalities, the strict isolation measures, and the closure of schools and universities across many countries placed significant psychological strain on society, patients, healthcare workers, and the elderly. Even if most individuals were not directly infected, they were exposed to the psychological effects of the pandemic. The prolonged emotional tension, which persisted both during and immediately after the crisis, had adverse consequences for both physical and mental health. The negative effects also included weakened immune function, which was particularly relevant in the context of an infectious disease outbreak [40].

The COVID-19 pandemic significantly disrupted routine immunization programs around the world, with both direct and indirect public health consequences (Table 2). Global data indicated that over 68 countries experienced moderate to severe interruptions in routine vaccination services, with more than 80 million children at risk of vaccine-preventable diseases due to missed immunizations [41,42]. These disruptions were not only due to overwhelmed health systems but also influenced by parental fear of SARS-CoV-2 exposure, lockdown policies, redeployment of healthcare workers, and logistical barriers to vaccine distribution [41].

Low- and middle-income countries were particularly vulnerable, as they faced compounded challenges such as weak healthcare infrastructure, political instability, and reduced access to essential services. Immunization campaigns for diseases such as measles, polio, diphtheria, and rubella were suspended or postponed in dozens of countries. This situation has raised serious concerns about the resurgence of controlled or eliminated diseases. For example, delays in measles vaccination were associated with outbreaks in regions already experiencing fragile healthcare systems [42].

The pandemic also highlighted inequalities in vaccine access and coverage. In some countries, there was a marked decline in the number of administered vaccines—for instance, up to a 63% drop in vaccinations among children over age two in certain areas of the United States during the initial months of the pandemic [43].

International organizations such as WHO and UNICEF issued strong recommendations to resume and continue routine immunization efforts wherever feasible, stressing that the benefits of maintaining vaccination coverage outweigh the potential risks of SARS-CoV-2 transmission during clinic visits [41,42,44]. Additionally, it was noted that live attenuated vaccines—such as oral polio vaccine and BCG—may offer non-specific immune-boosting effects, possibly enhancing resistance to unrelated pathogens, including SARS-CoV-2, though this remains under investigation [41,45,46]. This experience underscores the critical importance of strengthening immunization infrastructure and public trust during pandemics. Strategic planning should include catch-up vaccination campaigns, risk communication, and system resilience to ensure that routine preventive health measures are not compromised during future crises [41].

While physical distancing helped slow the spread of the virus, this practice, by its very nature, restricted social interactions in physical spaces, potentially weakening social connectedness. The reduced availability of in-person interactions raised concerns, as long-term scientific studies have demonstrated the fundamental importance of social relationships for individual well-being. Due to the potentially negative effects of the COVID-19 pandemic and its impact on well-being, researchers in social, behavioral, and clinical sciences issued urgent calls for action to mitigate the pandemic’s effects. One of the primary concerns highlighted by researchers was the risk of increased social isolation and strain on intimate relationships, which could be exacerbated by various stressors (e.g., social, economic, and health-related stress) associated with the pandemic. However, it is important to note that physical distancing, which allows interaction with household members, facilitates digital communication, and applies to entire communities rather than individuals, is not equivalent to social isolation [47].

The psychological consequences of the COVID-19 pandemic were observed worldwide. A study conducted in the United States, which analyzed individual experiences from January 2020 (*n* = 1010) to June 2020 (*n* = 3020), reported one of the largest declines in happiness and life satisfaction, along with deterioration in mental and physical health, as well as moderate declines in life meaning and overall well-being [48]. Similarly, a longitudinal study in the United Kingdom, tracking approximately 2000 respondents from June 2019 to June 2020, found that positive emotions (e.g., happiness, energy, inspiration, optimism, satisfaction) became less common, while some negative emotions (e.g., sadness, stress, fear, frustration) intensified during the initial outbreak in March. However, most individuals eventually returned to pre-pandemic emotional levels during the lockdown in May [49]. Interestingly, other negative emotional states, such as loneliness and apathy, decreased, while some remained stable (e.g., boredom) in the first month of the outbreak but began to increase as the lockdown progressed.

Although the negative psychological effects of the COVID-19 pandemic were clear, some individuals demonstrated remarkable resilience. In France, researchers conducted three survey waves between 1 April and 6 April 2020 and found that respondents, particularly those with limited direct exposure to the disease, reported improvements in health and well-being during quarantine, regardless of income level [50]. Other studies reported no changes in life satisfaction before and during the pandemic. In a sample of adults primarily from the United States and the United Kingdom (*n* = 336), respondents did not perceive any changes in life satisfaction between mid-February and late May 2020 [47,51].

The occurrence of severe infectious diseases in the past has contributed to heightened societal fear, as confirmed by research on previous epidemics. Studies examining the psychological impact of severe acute respiratory syndrome (SARS), Middle East respiratory syndrome (MERS), and H1N1 (influenza A virus subtype H1N1) influenza demonstrated clear associations between pandemic-related fear and symptoms of post-traumatic stress disorder (PTSD), anxiety, and depression [52,53]. The findings from these previous outbreaks revealed a wide range of psychosocial effects, both on individual and societal levels. Severe anxiety, distress, fear, and heightened emotional tension were common reactions within Chinese society during the 2003 SARS epidemic. During this outbreak, numerous studies focused on the psychological effects on non-infected populations, identifying significant mental health concerns, particularly among younger individuals and those experiencing heightened guilt [40].

**Table 2:** Expanded impact of COVID-19 on routine immunization and public health.

Area of Impact	Key Observations	Source
Disruption of immunization services	Over 68 countries experienced moderate to severe disruptions; 80 million+ children at risk of missing essential vaccines	Dinleyici et al. [42]
Causes of interruption	Lockdowns, parental fears, workforce reallocation, transport and logistics barriers	Dinleyici et al. [42]
Vaccine-preventable disease resurgence	Suspension of campaigns for measles, polio, diphtheria, etc.; outbreaks already observed in countries like Pakistan, Venezuela, and Nepal	Dinleyici et al. [42]
Decline in vaccine uptake	US data showed drops up to 63% in children > 2 years old; global trend of missed doses	Santoli et al. [44]
Recommendations from global agencies	WHO and UNICEF stressed the need to maintain or resume routine immunizations; emphasized risk–benefit balance	WHO [45]
Live vaccines and non-specific immunity	Hypothesized that BCG and oral polio vaccines may offer cross-protection via trained innate immunity	Chumakov et al. [46]
Vaccine hesitancy during the pandemic	Anti-vaccine misinformation spread rapidly; reduced trust in immunization programs may affect future uptake	Dinleyici et al. [42]
Postponed campaigns and outbreaks	Multiple outbreaks of measles and polio followed campaign suspensions; 178 million people at risk of missing measles vaccine	WHO [46]
Disparities in low-income countries	LMICs faced the greatest disruption due to weak health systems and conflict zones, with measles resurgence reported	Roberts et al. [43]; Hoffman et al. [54]
Protective potential of existing vaccines	BCG and OPV may offer non-specific protection through trained immunity, but evidence remains inconclusive	Chumakov et al. [46]
Call for catch-up programs	WHO recommends enumerating children who missed doses and developing customized catch-up vaccination plans	WHO [45]

## 7. Impact of Nutritional Status

Most studies have shown that approximately 40–50% of participants increased their food intake during the COVID-19 pandemic and snacked more frequently. This trend was even more pronounced among overweight and obese individuals, suggesting that pre-existing unhealthy habits were more likely to intensify. Similarly, a significant increase in alcohol consumption was observed among individuals with alcohol dependence, compared to the general population (64% vs. 14%), and more than 45% of smokers reported smoking more. Therefore, particular attention should be given to lifestyle changes in high-risk groups where unhealthy habits are already well-established.

Additionally, during the first wave of the COVID-19 pandemic, the number of daily meals consumed increased significantly, although no substantial changes were observed six months after the outbreak. In terms of dietary composition, most studies indicated a decrease in the consumption of fast food, instant soups, sugary beverages, and energy drinks, while consumption of sweets, eggs, potatoes, canned meats, and alcohol increased. However, some studies reported a decrease or no change in alcohol consumption.

Furthermore, an increase in water, fruit, vegetable, and nut consumption was observed among Polish primary school teachers and Serbian university students. The most notable changes in dietary patterns were observed among individuals who feared COVID-19 infection and those who strictly adhered to isolation measures. Interestingly, most respondents did not experience food accessibility issues, but more than 40% of individuals who shifted to a less healthy diet reported such difficulties during the pandemic. Adherence to nutritional recommendations was lowest among individuals struggling with stress and psychological burdens, indicating that this group was most vulnerable to worsening dietary habits during the pandemic. Problematic eating behaviors were identified in over 14% of Polish adults, as assessed by the Yale Food Addiction Scale 2.0 [55].

### 7.1. The Importance of Vitamins and Macronutrients in Immunity

**Figure 2:**
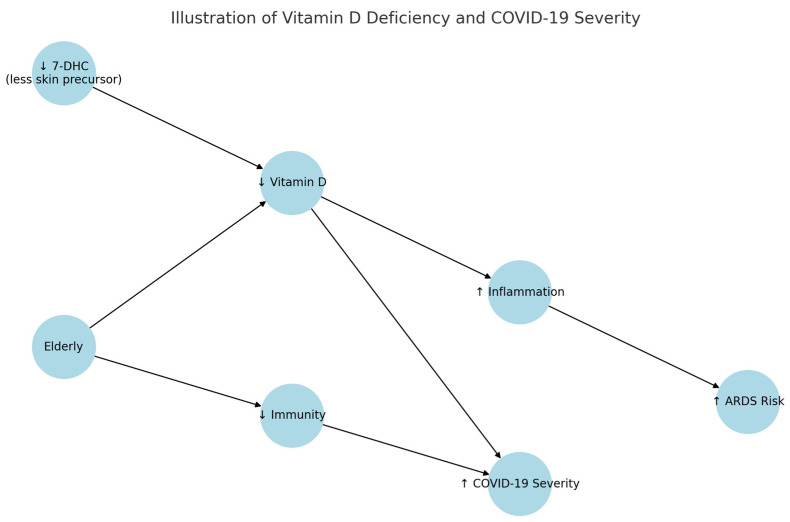
The diagram above illustrates the relationships between reduced levels of 7-DHC (a precursor of vitamin D), vitamin D deficiency, increased inflammation, and the risk of ARDS, as well as the impact of aging on immunity and the severity of COVID-19. Explanation: up arrow, higher content, down arrow lower concentration.

Vitamin D has been strongly associated with multiple risk factors related to COVID-19 (Figure 2, Table 3). Its deficiency has been linked to advanced age, obesity, male sex, hypertension, residence in colder climates, and coagulation disorders, which are, in turn, associated with poorer treatment outcomes. With age, the concentration of active vitamin D declines due to reduced sunlight exposure and decreased 7-dehydrocholesterol (7-DHC) production in the skin. This may partially explain the higher COVID-19 mortality observed in the elderly. This population also exhibited a shift in immune function towards a pro-inflammatory state known as “inflame-aging”, leading to chronic low-grade inflammation, cumulative biological damage, and the progression of chronic diseases. Studies have shown that vitamin D increases anti-inflammatory cytokines and reduces pro-inflammatory cytokines in older individuals. This immunomodulatory effect proved particularly beneficial during cytokine storms, which is characteristic of COVID-19 patients with acute respiratory distress syndrome (ARDS). A meta-analysis of eight observational studies involving 20,966 individuals demonstrated that low vitamin D levels were associated with an increased risk of pneumonia [56].

Recent research has demonstrated a correlation between vitamin D deficiency and the severity of COVID-19. Observational studies have shown that low serum 25(OH)D levels are associated with higher mortality and prolonged hospitalization. However, findings from randomized controlled trials (RCTs) remain inconsistent. For example, a 2021 RCT by Murai et al. involving a single high dose of vitamin D3 showed no significant reduction in hospital stay duration among patients with moderate to severe COVID-19. Other studies, however, report potential improvements in inflammatory markers and reduced symptom severity. This ongoing debate highlights the need for further high-quality, placebo-controlled trials to determine the precise role of vitamin D supplementation in COVID-19 prevention and treatment [8,9,10,12,57].

A small study observed improvements in inflammatory biomarkers and selected respiratory parameters following intravenous administration of vitamin C. In one case, where a patient was treated with high doses of vitamin C after developing ARDS, it was noted that she was weaned off mechanical ventilation within five days. However, it should be considered that she also received antiviral medications simultaneously. There is evidence that vitamin C played a crucial role in cases of secondary sepsis following pneumonia, a phenomenon also observed in COVID-19. Additionally, unpublished data from a study conducted on 50 patients in China suggested the beneficial effects of high-dose vitamin C administration in severe cases of the disease, although these findings require further confirmation. For this reason, vitamin C supplementation appears to be a reasonable approach for individuals with micronutrient deficiencies who are at risk of COVID-19 infection. It may support immune responses and help prevent severe disease progression [56].

Due to its immunomodulatory and antiviral properties, zinc has been considered a potential adjunct therapy for COVID-19 patients. It has been suggested that zinc supplementation could enhance the efficacy of other therapeutic approaches, such as hydroxychloroquine. Observations of four COVID-19 patients who received high doses of zinc reported clinical improvement in symptoms. Research indicated that zinc supplementation might alleviate symptoms such as lower respiratory tract infections, attributed to its ability to inhibit viral binding and replication, which could be particularly relevant in the context of COVID-19. During the pandemic, emphasis was also placed on the importance of maintaining adequate levels of vitamins C, D, and E to reduce symptom severity and shorten the duration of respiratory infections. Scientific evidence highlighted the role of minerals such as zinc in enhancing immune responses and inhibiting viral replication. Adequate dietary intake of these nutrients was considered essential for proper immune system function. Fruits, vegetables, meat, fish, poultry, and dairy products were identified as good sources of vitamins and minerals. The benefits of increased consumption of vitamins D, C, E, zinc, and omega-3 fatty acids in supporting immune functions during illness were also emphasized. However, it was noted that many studies on the use of these nutrients in COVID-19 patients utilized doses too high to be obtained exclusively from diet. Supplementation with higher doses of these nutrients during infection yielded positive outcomes, and given their low-risk profile, it was considered a reasonable strategy. However, further research is needed to determine the effective doses of vitamins C, D, E, zinc, and omega-3 fatty acids in COVID-19 prevention and symptom management [54].

Due to its immunomodulatory and antiviral properties, zinc has gained attention as a potentially beneficial supplement for COVID-19 patients. Zinc deficiency is associated with increased levels of pro-inflammatory cytokines and disruption of lung epithelial barrier function, processes that may exacerbate disease severity [7,57]. Research shows that zinc supports innate and adaptive immunity by promoting neutrophil recruitment, enhancing natural killer (NK) cell activity, phagocytosis, oxidative burst, and the activation of CD4^+^ and CD8^+^ T cells. Zinc supplementation has been shown to restore T cell and NK cell counts and increase interleukin-2 (IL-2) and its soluble receptor expression. Moreover, zinc may directly inhibit the replication and transcription of coronaviruses, interfering with viral protein synthesis [8,11,57,58]. Clinical observations, including a case series involving high-dose zinc supplementation, suggest symptomatic improvement in COVID-19 patients [9,12,57]. A registered clinical trial in Australia is also investigating intravenous zinc administration in COVID-19-positive individuals [59]. However, while promising, further randomized trials are needed to confirm these benefits fully.

Omega-3 fatty acids, such as eicosapentaenoic acid (EPA) and docosahexaenoic acid (DHA), also play a role in modulating inflammatory responses and supporting immune function. These polyunsaturated fatty acids are known to inhibit the replication of some viruses, including influenza, and have been proposed to improve oxygenation in patients with respiratory distress [57,60]. Nonetheless, evidence remains mixed. While the European Society for Parenteral and Enteral Nutrition has noted their potential, some studies have warned of increased oxidative stress in certain contexts due to membrane susceptibility [61]. Until conclusive clinical data are available, the use of omega-3 supplements, particularly in high doses, should be approached with caution in COVID-19 patients.

**Figure 3:**
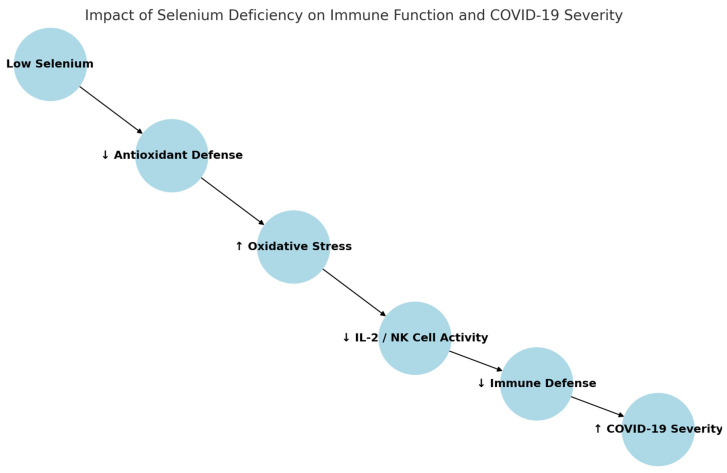
Mechanistic pathway linking selenium deficiency to immune suppression and severe COVID-19. Explanation: up arrow, higher content, down arrow, lower concentration.

Selenium, a trace element with potent antioxidant properties, plays an important role in regulating immune responses and reducing oxidative stress (Figure 3). Its deficiency has been associated with altered immune cell activity, increased susceptibility to viral infections, and more severe disease outcomes. Epidemiological studies from China suggest a correlation between regional selenium levels and COVID-19 recovery rates [62,63,64,65]. Selenium acts synergistically with vitamin E to enhance T-cell proliferation, increase interleukin-2 (IL-2) secretion, and boost natural killer (NK) cell activity, collectively contributing to a stronger antiviral response [63,64,65,66]. While direct evidence on selenium supplementation in COVID-19 remains limited, these findings highlight the potential role of maintaining adequate selenium levels to support immune defense mechanisms. Further clinical research is needed to evaluate selenium’s therapeutic efficacy in COVID-19 patients.

An interesting phenomenon observed during the COVID-19 pandemic was the increased consumption of products believed to support immunity, such as garlic, ginger, honey, lemon, raspberry syrup, turmeric, and fermented fruits and vegetables. Additionally, during the first two waves of the pandemic, there was a rise in the intake of dietary supplements containing vitamins C and D, zinc, and omega-3 fatty acids—nearly 35% of Polish adults reported taking at least one new supplement, and almost 23% reported increased spending on supplements [54]. Similar findings were observed among Serbian medical students, of whom over 40% increased their overall supplement intake, particularly those containing vitamins C and D and zinc [66]. However, studies conducted over a year after the pandemic outbreak in Poland found no changes in the consumption of vitamin C, vitamin D, or magnesium supplements. A possible explanation for this phenomenon is the initial fear of COVID-19, which drove individuals to increase immune-supporting supplementation. This trend later subsided, as partially suggested by studies by Hamulka et al. [67]. Another factor could be the perceived safety associated with the rollout of vaccination programs, which reduced the perceived necessity for supplements. Notably, individuals with greater nutritional knowledge had a higher index of health-conscious diets in March 2020, at the beginning of the pandemic. However, by October 2020, no significant statistical associations were observed, suggesting a lack of consistency in maintaining healthy dietary habits. In this context, the role of nutrition-supporting applications should be appreciated, as they contributed to positive dietary changes during the COVID-19 pandemic.

Several randomized controlled trials (RCTs) have investigated the effects of vitamin D supplementation on respiratory infections, including COVID-19, with mixed results [10]. For example, a 2021 RCT by Murai et al. [10] found no significant impact on hospital stay duration in COVID-19 patients, though others have reported improved inflammatory markers.

The impact of the COVID-19 pandemic was particularly evident among students. More than 50% of undergraduate students reported that the pandemic negatively affected their eating habits, and 72–76% stated that it had an adverse impact on their physical activity. On the other hand, positive changes were noted by 30% of students regarding dietary habits and by 20% concerning physical activity. A more optimistic perspective was presented by Hoffmann et al. [66], who found that nearly 20% of respondents, primarily young adults, reported adopting a healthier diet during the second wave of COVID-19 in Poland, and 3% completely gave up stimulants, while only 11% admitted to less healthy eating habits. Similar results were obtained by Hamulka et al. [66], who demonstrated that 27.6% of respondents improved their diet, while 19.4% developed less favorable dietary habits [54,66].

### 7.2. The Relationship Between Nutritional Deficiencies and the Course of COVID-19

Researchers from University College London analyzed nearly 500 COVID-19 cases in patients with an average age of 68 years and found that abnormal sodium levels in the blood significantly increased the risk of mortality. It was determined that patients with low sodium levels were twice as likely to require advanced respiratory support, while those with elevated sodium levels had a threefold higher risk of death compared to those with normal sodium concentrations. According to Tzoulis, who led the study, sodium measurement could provide clinicians with valuable insights into which COVID-19 patients are at the highest risk of clinical deterioration or death. These findings may influence decisions regarding hospitalization and intensive care unit (ICU) admission (Table 4).

Experts emphasized that sodium levels are routinely checked upon patient admission to hospitals, and such testing is relatively inexpensive, with the potential for correcting imbalances easily. It was noted that elevated sodium levels could result from factors such as diarrhea, vomiting, or inadequate fluid intake. Sodium is also a major component of table salt. Another essential element that may play a significant role in COVID-19 progression is zinc [57].

Scientists from Pompeu Fabra University in Spain conducted a study on a group of 249 patients with an average age of 65 years. The results demonstrated that low zinc levels were often associated with increased inflammation and an average threefold longer hospital stay. Among patients with low zinc levels, one in five died, whereas in the higher zinc group, the mortality rate was only 5%. Survivors had an average zinc concentration of 62 µg/dL, whereas those who died had a significantly lower zinc level of 49 µg/dL [68].

A growing body of evidence supports the significant role that micronutrient status plays in modulating COVID-19 disease severity. In a prospective study by Voelkle et al. [69] conducted in Switzerland on 57 hospitalized COVID-19 patients, it was found that 79% of the patients had at least one micronutrient deficiency, and 33% had three or more deficiencies. Among the most prevalent deficiencies were selenium (51%), vitamin D (40%), vitamin A (39%), and zinc (39%). The authors also observed that a higher number of deficiencies correlated with a greater likelihood of intensive care unit (ICU) admission and mechanical ventilation, with patients suffering from multiple deficiencies experiencing longer hospital stays on average [68].

Interestingly, while vitamin A and zinc deficiencies were associated with significantly worse clinical outcomes—including a more than sevenfold increase in the risk of ICU admission or in-hospital mortality—selenium deficiency, though common, did not reach statistical significance in predicting adverse outcomes in this cohort. However, this contrasts with findings from other countries, such as China and Belgium, where low selenium status has been linked to increased COVID-19 mortality [65,69].

The Swiss study also highlighted that certain micronutrients showed positive intercorrelations—for example, vitamin D was moderately correlated with both vitamin A and selenium, while selenium positively correlated with folic acid. These patterns suggest that micronutrient deficiencies may not occur in isolation and should be addressed holistically. The study supports the notion that evaluating and correcting micronutrient deficiencies, including selenium, could be a valuable component of personalized COVID-19 management strategies. Nevertheless, randomized trials are needed to determine whether supplementation translates into clinically meaningful improvements in outcomes [68].

In a study conducted by Im et al. [70], the most frequent deficiencies observed in COVID-19 patients were vitamin D and selenium deficiencies. Moreover, nearly all patients experiencing respiratory distress were classified as malnourished. It remains unclear whether nutrient deficiencies directly affected immunity or merely exacerbated the patient’s overall health status. However, given the growing evidence pointing to excessive inflammatory responses as a key factor in severe COVID-19 progression, particular attention should be given to vitamin D and selenium deficiencies [55,57].

Vitamin D supports the production of antimicrobial peptides in the respiratory epithelium, which reduces the risk of viral infections and mitigates symptom severity. Renin-angiotensin system dysregulation is one of the primary mechanisms of lung damage in COVID-19, and vitamin D also plays a role in modulating anti-inflammatory mediators, which is particularly relevant in preventing excessive inflammatory responses caused by SARS-CoV-2 infection. These mechanisms suggest that individuals with vitamin D deficiency may be at higher risk of infection and severe disease progression [70].

In another study, a group of COVID-19 patients exhibited a high prevalence of vitamin D deficiency compared to a control group. However, vitamin D supplementation yielded inconsistent benefits in treating or preventing most diseases, except for rickets and osteomalacia [71]. An important exception to this general trend is upper respiratory tract infections. A 2017 meta-analysis demonstrated that vitamin D supplementation may protect against acute respiratory infections.

In the study, even the control group showed a 43.3% prevalence of vitamin D deficiency, making it difficult to establish a direct link between vitamin D deficiency and increased infection risk. Nonetheless, considering the high mortality rates in long-term care facilities and countries at higher latitudes, along with the pathophysiology of COVID-19, it is believed that vitamin D deficiency may worsen severe COVID-19 outcomes. Further research is necessary to determine whether vitamin D supplementation could aid clinical progress in COVID-19 patients [72].

The potential benefits of vitamin D supplementation in the prevention and management of COVID-19 remain a subject of debate. While some researchers advocate for widespread supplementation due to its immunomodulatory properties, others emphasize the lack of definitive clinical evidence. Given the variability in study results, it is crucial to approach vitamin D supplementation with an evidence-based perspective. Health authorities generally recommend maintaining adequate vitamin D levels through sun exposure, dietary sources, or supplementation, particularly in populations at risk of deficiency. Future studies should focus on large-scale, placebo-controlled trials to determine the precise impact of vitamin D on COVID-19 disease progression [73,74,75].

**Table 4:** Characteristics and outcomes of studies analyzing the role of vitamins and minerals in COVID-19.

Study/Author	Population/Country	Deficiency Observed	Main Findings
Radujkovic et al. [8]	Hospitalized, Germany	Vit D	21% mortality vs. 3.1% in sufficient group
Voelkle et al. [69]	Hospitalized, Switzerland	Selenium, Vit D, Vit A, Zinc	More deficiencies → longer stay and ICU
Im et al. [70]	COVID-19 patients, Korea	Vit D, Selenium	Most with respiratory distress were malnourished
Hamulka et al. [67]	Polish adults	Inconsistent supplement use	Fear-related spike, later decline
Zhang et al. [62]	China	Selenium	Higher selenium areas had better survival

## 8. Impact of the Pandemic on Society

Studies conducted in 2020 by Długosz et al. [73] indicate that Poles generally perceive the overall impact of the COVID-19 pandemic as negative, highlighting the economic crisis and increasing social inequalities. Only a small fraction of respondents recognized any positive aspects of the pandemic.

Older individuals and those with higher education levels were more likely to perceive the negative effects of the pandemic. Similarly, individuals dissatisfied with their lives evaluated its impact on society more critically compared to those who were satisfied or undecided. Respondents with liberal views predominantly rated the pandemic’s impact as negative, whereas those with conservative views were more likely to declare a positive influence. Individuals who negatively assessed their financial situation were also more inclined to highlight the negative consequences of the pandemic.

Regarding expectations for the future of the pandemic, opinions were divided. Approximately one-third of respondents believed that the worst was yet to come, another third predicted no change, and only a small fraction expected improvement. Pessimism increased with age, as older individuals were more likely to anticipate worsening conditions, whereas younger respondents were more optimistic, believing that the worst had already passed.

Education level also played a role in these assessments. Respondents with higher education were more likely to predict that society would face difficult times, whereas those with lower education levels were more likely to believe in future improvements. Individuals satisfied with their lives mostly expected the situation to remain stable, while dissatisfied respondents more often feared further deterioration.

Among those with a high sense of threat, pessimism was more prevalent, whereas those with a moderate or low sense of threat predominantly believed that the epidemic situation would remain unchanged [73,74,75].

The findings suggest that future pandemic preparedness should prioritize rapid diagnostics integrated with population-level nutritional screening and psychosocial interventions. This review underscores the value of a holistic framework that simultaneously addresses biomedical, behavioral, and systemic resilience.

## 9. Conclusions

The COVID-19 pandemic underscored the necessity of early diagnostics, optimal nutritional status, and psychosocial support in the pandemic response. Key findings point to the value of micronutrient sufficiency, especially vitamin D and zinc, in reducing disease severity. The experience highlighted the need for integrated public health approaches that include nutritional screening, personalized supplementation, and coordinated epidemiological strategies to enhance healthcare system resilience.

## Figures and Tables

**Table 1:** Summary of serological and molecular tests for COVID-19 diagnosis.

Test Type	Target	Sensitivity	Time to Result	Notes
RT-PCR	Viral RNA	High	Several hours	Gold standard, requires lab
Rapid Antigen	Viral proteins	Moderate	15–30 min	Useful for symptomatic screening
ELISA/CLIA	IgG, IgM antibodies	Variable	1–3 h	Used for seroprevalence
LFIA	IgG, IgM antibodies	Moderate	15–30 min	Point-of-care serology
FET Biosensor	Virus activity	Very high	Rapid	Experimental, high sensitivity

**Table 3:** Studies on nutrients and immunity.

Nutrient	Mechanism in Immunity	COVID-19 Impact	Sources
Vitamin D	Regulates cytokine response	Linked to ARDS, inflammatory control, low levels linked to higher severity	Sunlight, fatty fish
Vitamin C	Antioxidant, supports epithelial barrier	Reduces severity of ARDS, improves biomarkers, sepsis	Citrus fruits, vegetables
Zinc	Supports T cells and NK cells	Inhibits viral replication, enhances IL-2, linked to better outcomes	Meat, legumes
Selenium	Antioxidant, NK cell activation	Correlated with recovery rates, viral defense, deficiency associated with worse prognosis	Brazil nuts, fish
Omega-3	Anti-inflammatory	Improves oxygenation, potential risks in excess	Fish oils, flaxseeds
